# Effect of surface-partial-volume correction and adaptive threshold on segmentation of uroliths in computed tomography

**DOI:** 10.1371/journal.pone.0286016

**Published:** 2023-06-23

**Authors:** Jakob Neubauer, Konrad Wilhelm, Christian Gratzke, Fabian Bamberg, Marco Reisert, Elias Kellner

**Affiliations:** 1 Department of Diagnostic and Interventional Radiology, Faculty of Medicine, Medical Center – University of Freiburg, Freiburg, Germany; 2 Department of Urology, Faculty of Medicine, Medical Center – University of Freiburg, Freiburg, Germany; 3 Medical Physics, Faculty of Medicine, Medical Center – University of Freiburg, Freiburg, Germany; 4 Department of Stereotactic and Functional Neurosurgery, Medical Center – University of Freiburg, Freiburg, Germany; Chung-Ang University Gwangmyeong Hospital, REPUBLIC OF KOREA

## Abstract

Computed tomography (CT) is used to diagnose urolithiasis, a prevalent condition. In order to establish the strongest foundation for the quantifiability of urolithiasis, this study aims to develop semi-automated urolithiasis segmentation methods for CT images that differ in terms of surface-partial-volume correction and adaptive thresholding. It also examines the diagnostic accuracy of these methods in terms of volume and maximum stone diameter. One hundred and one uroliths were positioned in an anthropomorphic phantom and prospectively examined in CT. Four different segmentation methods were developed and used to segment the uroliths semi-automatically based on CT images. Volume and maximum diameter were calculated from the segmentations. Volume and maximum diameter of the uroliths were measured independently by three urologists by means of electronic calipers. The average value of the urologists´ measurements was used as a reference standard. Statistical analysis was performed with multivariate Bartlett’s test. Volume and maximum diameter were in very good agreement with the reference measurements (r>0.99) and the diagnostic accuracy of all segmentation methods used was very high. Regarding the diagnostic accuracy no difference could be detected between the different segmentation methods tested (p>0.55). All four segmentation methods allow for accurate characterization of urolithiasis in CT with respect to volume and maximum diameter of uroliths. Thus, a simple thresholding approach with an absolute value may suffice for robust determination of volume and maximum diameter in urolithiasis.

## Introduction

Urolithiasis is a common condition that has shown a marked increase in prevalence over the last 40 years [1, 2]. Ultrasound, radiography and computed tomography (CT) are available for the imaging diagnosis of urolithiasis. Compared to the other two methods, CT has a higher diagnostic accuracy for the detection of uroliths [3–5]. In addition, the size of the uroliths can be determined most accurately with CT [6] and is independent of the type of CT scanner and reconstruction algorithm [7]. This holds true for low-dose examinations as well [8].

The exact urolith size is important for therapy planning (e.g. ureterorenoscopic treatment vs. percutaneous treatment with standard shaft size or miniaturized approaches) and for correct allocation of patients to study groups [9]. However, there is no uniformly accepted method for determining the size of uroliths in CT. Often stone size is determined by manually measuring the maximum diameter in planar CT reconstructions in one or two planes. However, it was shown that stone diameter does not necessarily correlate with the real stone burden [10]. Especially for stones with irregular shapes, the size is not accurately captured by the diameter. Therefore, stone burden should rather be expressed in volume. This is further supported by the fact that measurements of stone volume are more reliable than measurements of stone diameter [10] and have been shown to be of value in preoperative planning [11, 12]. Stone volume is also able to predict success of therapy [13, 14]. The volume can be estimated from the diameter with approximation formulas, but inter-reader reliability of this method is low [15]. Much more accurate for the determination of the stone volume are methods which segment the uroliths in the CT data sets [16]. Typically a region growing algorithm is used, which uses either a fixed cut-off value [17, 18] or a significant jump in density values as a limit [19].

**Fig 1:**
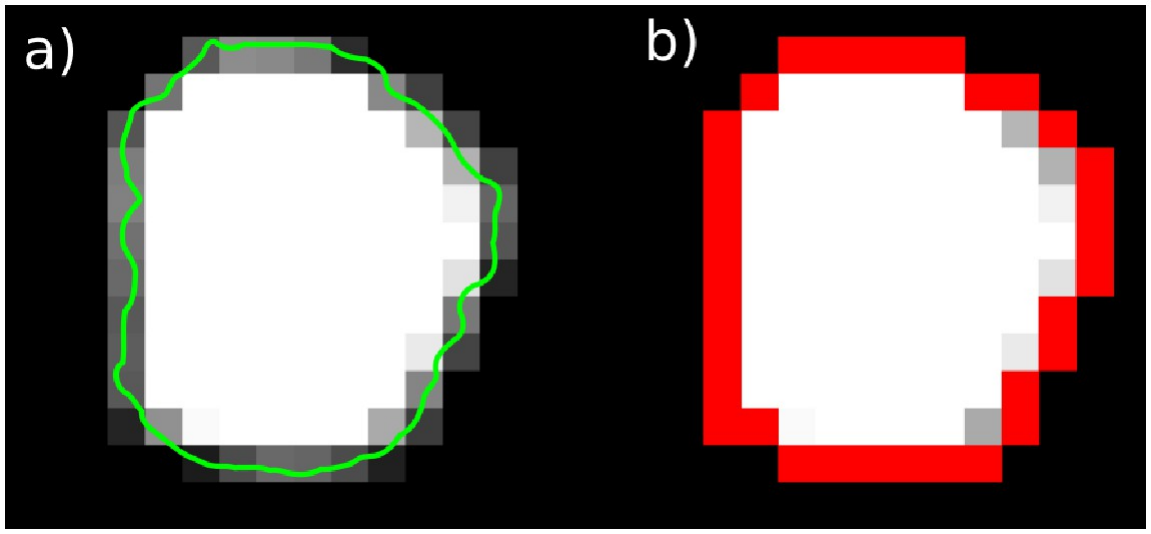
Surface partial volume effect. When imaging a real-world object (green outline) with a limited spatial resolution, pixels on the surface contain a mixture of object and the surrounding (“partial volume effect”). This effect is further enhanced by additional blurring inherent to CT scans. We introduced an approach where the objects are segmented by first using a relatively low HU threshold, and subtracting surface pixels (red) multiplied with a global factor, see text.

**Fig 2:**
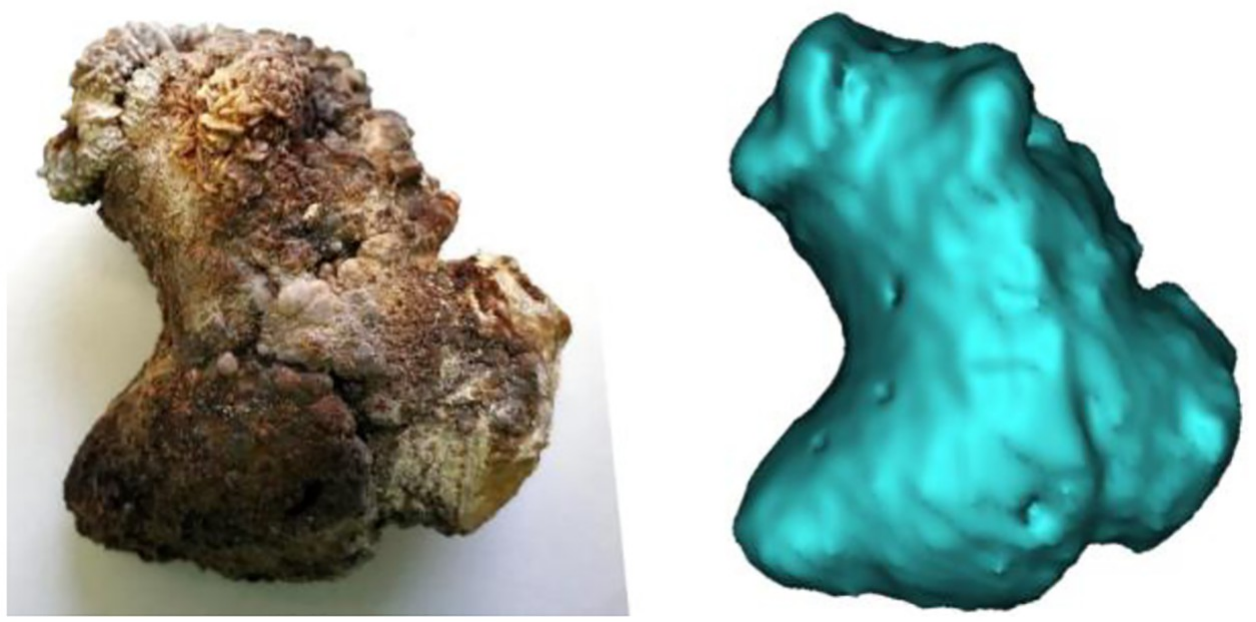
Segmentation-derived 3D-volume rendering and photograph of an urolith. The basic shape of the urolith is reproduced accurately, but the fine structure of the surface is not comprehensible in the 3D representation.

Until now, however, it has not been investigated whether there is a difference between the individual algorithms and if so, which algorithm has the highest diagnostic accuracy. Also, challenges of segmentation may be attributed to adequate estimation of partial volume effects on the stone surface (Figs 1 and 2) and to different densities of uroliths [12]. Thus, in the present study, we develop different algorithms with appropriate correction mechanisms and compare these algorithms with respect to diagnostic accuracy in terms of volume and maximum stone diameter.

## Materials and methods

The study was approved by the ethics committee of the University of Freiburg. Since we only used fully anonymized patient and animal data, informed consent was waived by this committee. We included 101 urinary stones from a urolithiasis reference center, which were larger than 4 mm in minimum diameter.

Uroliths were composed of calciumoxalate-monohydrate (2), calciumoxalate-dihydrate (6), brushite (18), cystine (2), struvite (2), uric acid (8). In 41 cases stone composition was not clear (not assessed). Sixtyseven of the stones originated from humans. Due to data protection issues the baseline characteristics of the patients could not be obtained from the donating stone center. Since human stones bigger than 5–6 mm are not available due to the current extraction techniques, we added 34 animal stones (Canis lupus familiaris stones).

The uroliths were positioned in an anthropomorphic phantom. The phantom has previously been described in detail [10]. The phantom consisted of a large nonradiopaque polypropylene tub in size of the abdomen of an adult weighing about 70 kg (560 x 363 x 128 mm). The tub was completely filled with water. Human vertebral bodies were inserted into the dorsal part of the tub. In the place of imaginary kidneys, kidney stones were positioned on four small nonradiopaque polyvinyl chloride pedestals on each side. In order to be able to examine all stones on CT, the stones were replaced several times.

The phantom was prospectively examined in a CT (Siemens Somatom Flash; Siemens, Forchheim, Germany) with standard low dose settings: 100 kVp and 55 mAs, resulting in a CTDI_vol_ of 2.3 mGy. The images were reconstructed axially with a slice thickness of 0.7mm and a pixel spacing of 0.7 x 0.7mm and the I31f kernel. All uroliths were photographed. Three urologists with 4–7 years of experience independently measured the maximum diameter of the uroliths with a digital sliding caliper. They also determined the volume using the water displacement method as described in Jain et al. [19]. The mean values of the three measurements of the urologists were used as reference standard.

### Software-tool and algorithm

A semi-automated procedure for stone segmentation was implemented as a graphical user interface in the medical imaging platform NORA (www.nora-imaging.com, Medical Center—University of Freiburg). With the tool, circular annotation markers can be drawn around the uroliths, yielding a center point and maximum radius. Annotations were performed by a board approved radiologist with eight years of experience in uroradiology. Based on the annotations, the uroliths were segmented using four different approaches.

1. Fixed threshold in absolute HU units
  In this approach, an absolute, common threshold (P1) for all stones has been used. Parameters: Threshold (P1).
2. Fixed threshold in absolute HU, and additional surface-partial-volume correction
  Same as method 1, but with additional partial volume correction (see Table 1). PV correction was performed by subtracting the surface voxels multiplied with a factor. Parameters: Threshold (P1) and surface factor (P2).
3. Adaptable threshold scaling with median HU value of stone
  Each stone was roughly segmented by applying a relatively low threshold of 20 HU. Then, a stone-individual threshold was used based on the median value of this rough segmentation, multiplied with a factor (which was the same for all stones) Parameters: Factor for median multiplication (P1).
4. Adaptable threshold scaling with median HU and additional surface-partial-volume correction
  Same as method 3, but with additional partial volume correction. PV correction has been performed by subtracting the surface voxels multiplied with a factor. Parameters: Factor for median multiplication (P1) and surface factor (P2).

**Table 1:** Parameters and correlations coefficients for the four different algorithms.

Algorithm	Parameters		Volume			Diameter		
	P1	P2	ICC	difference (mL)	std (mL)	ICC	difference (mm)	std (mm)
**Absolute HU**	272 (5) HU	—	0.988 (0.033)	-0.036 (0.049)	0.23 (0.065)	0.994 (0.005)	0.011 (0.158)	0.79 (0.150)
**Absolute HU + PV correction**	194 (17) HU	0.29 (0.05)	0.990 (0.017)	0.014 (0.055)	0.23 (0.083)	0.995 (0.003)	-0.098 (0.149)	0.69 (0.105)
**Median HU**	0.48 (0.02) %	—	0.991 (0.011)	-0.020 (0.057)	0.24 (0.094)	0.995 (0.003)	-0.137 (0.145)	0.67 (0.100)
**Median HU + PV correction**	0.43 (0.04) %	0.11 (0.08)	0.989 (0.023)	0.004 (0.066)	0.26 (0.103)	0.995 (0.004)	-0.137 (0.143)	0.69 (0.100)

Optimal parameters, Intra-class correlation coefficient (ICC), mean differences and standard deviations of estimated volumes and diameters versus measured ground truth for all four methods. Parameters were optimized using the cross validation: Values are given as mean and standard deviation (in brackets) of 1,000 optimisation runs with random splits into training and validation sets. For the “Absolute HU” methods, the parameter P1 corresponds to the absolute threshold in Hounsfield units. For the “Median HU” methods, P1 corresponds to the percentage of stone-specific median-based thresholding. Parameter P2 corresponds to the relative amount of subtracted surface pixels for the partial-volume correction.

### Estimation of volumes and diameters from segmentations

The above described segmentation methods yield binary masks for any given set of parameters. In the first step, in order to account for potential cavities in the stones, a “fill-hole” step was applied to the masks. Then, volumes were calculated by multiplication of the voxel count with the volume per voxel. Diameters were calculated by calculating all pairwise distances between surface pixels and choosing the maximum value. This yields the distance between voxel centers, so an additional 0.7 mm (the size of one voxel) was added to account for the voxel edges.

### Threshold optimization

For all approaches, the optimal parameters P1 and P2 were searched with respect to minimizing a cost function. As a cost function, we used the following weighted mixture of the L1 norm for volumes and maximum diameters: 4| Ve- Vr | + | De—Dr | where Ve and De correspond to the estimated volumes and diameters, and Vr and Dr correspond to the reference ground truth values (For the ground truth reference, we used the mean ratings of the raters per stone). As a metric, we used the intraclass-correlation coefficient (ICC) as well as the mean difference and standard deviation of the reference minus estimated values. We performed parameter optimization by randomly splitting (1000 times) the total set of stones into a train set of size 80 and a validation set of size 21. Using the cost function above, Parameters P1 and P2 were optimized based on the training set, whereas the metrics (ICC, mean and std) were evaluated on the validation set. The procedure was repeated 1,000 times with different random splits into training and validation sets, and average (i.e. mean) and standard deviation of all repetitions was calculated. The average values can serve as average the final optimal parameters, and the standard deviation can be used to assess their robustness.

Finally, for visual assessment, correlation plots and Bland-Altman plots of the full set of stones were created by applying the average optimal parameters.

For demonstration purposes, 3D surface renderings of all stones were created by the NORA imaging viewer (www.nora-imaging.org) and are available in the supporting material.

### Statistical analysis

To compare the performance of the four different approaches, we performed a multivariate Bartlett’s test on the variances of the differences between reference standard and the method predictions with the null hypothesis of equal variances. A p-value under 0.05 was considered to indicate statistical significance.

## Results

**Fig 3:**
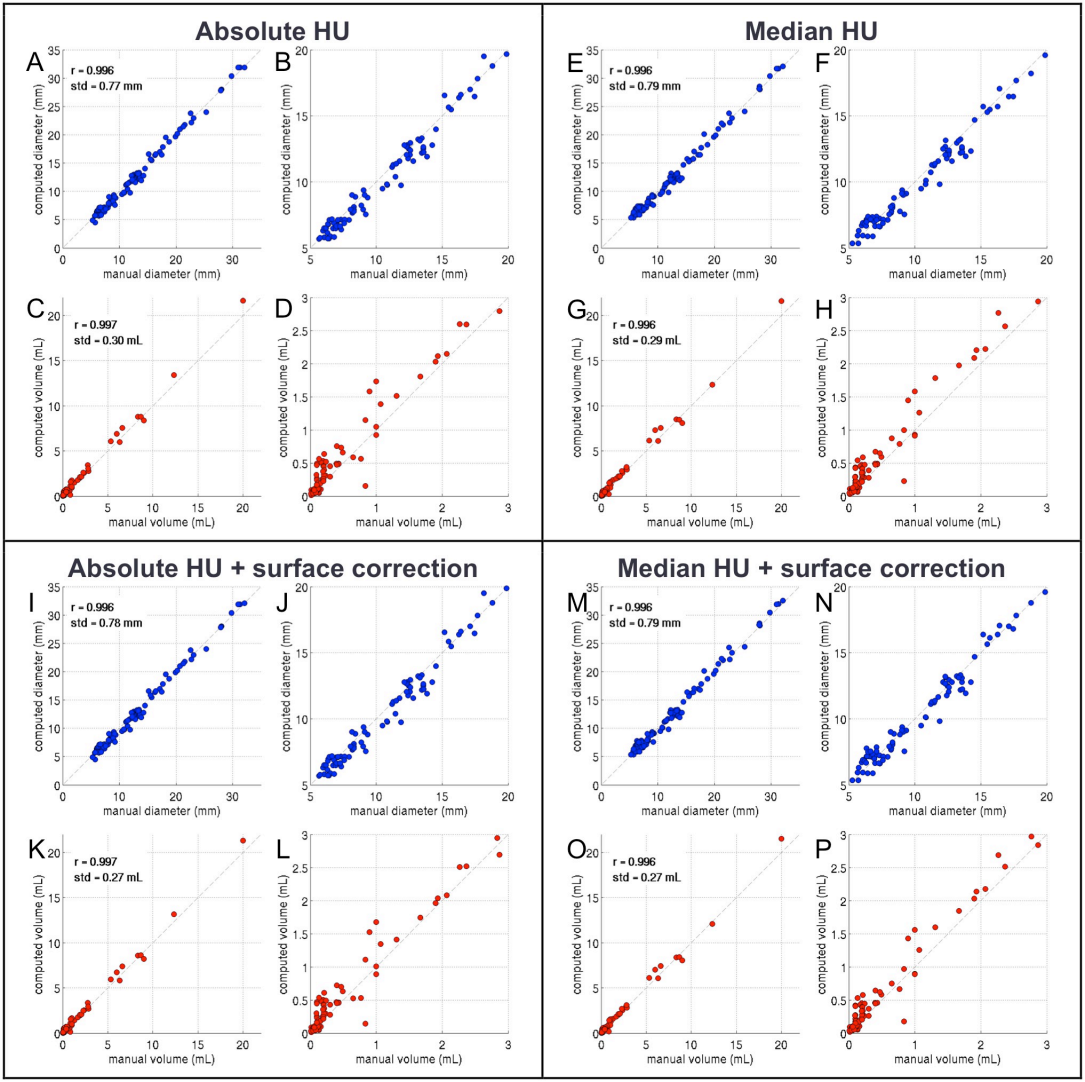
Correlation plots of estimated vs. reference standard diameters (A, B, E, F, I, J, M, N) and volumes (C, D, G, H, K, L, O, P) for all four segmentation approaches (A-D, E-H, I-L, M-P). The plots were created with all 101 uroliths using the optimal average parameters from the repeated optimization and validation procedure (see Table 1). Obviously, all approaches yield similar results, with good correlations.

**Fig 4:**
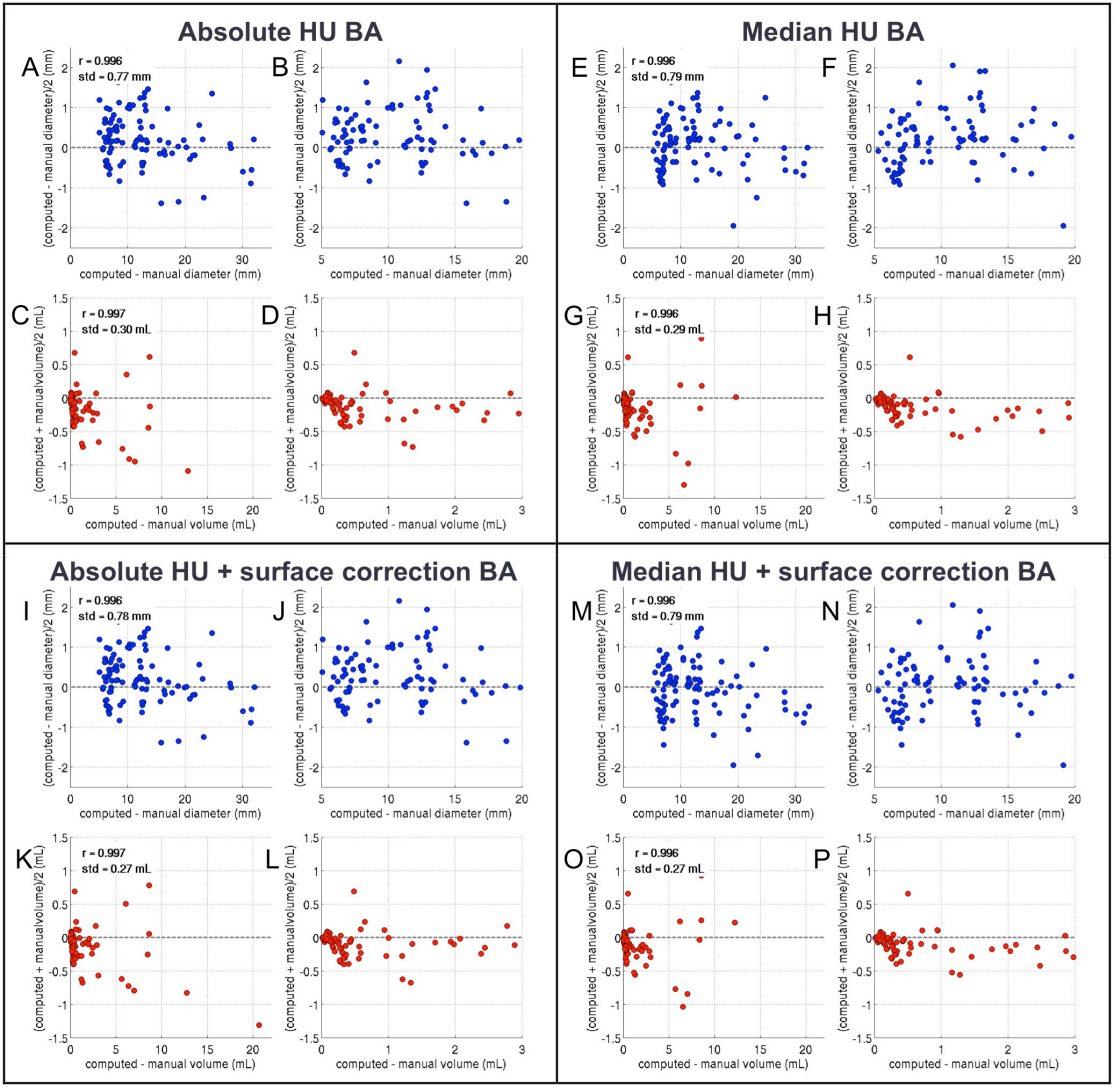
Bland-Altman plots of estimated vs. reference standard diameters (A, B, E, F, I, J, M, N) and volumes (C, D, G, H, K, L, O, P) for all four segmentation approaches (A-D, E-H, I-L, M-P). Dashed lines in Bland-Altman plots correspond to mean and 1.96* standard deviation. The plots were created with all 101 uroliths using the optimal average parameters from the repeated optimization and validation procedure (see Table 1). Obviously, all approaches yield similar results, with comparable mean differences and standard deviations.

Figs 3 and 4 show the agreement between stone sizes derived with the different algorithms, and the reference standard, both, as correlations and Bland-Altman-plots. Table 1 summarizes the optimal parameters, correlation coefficients, and standard deviations to the reference standard.

Correlation to the reference standard was excellent for all approaches (ICC>0.99). Also the other metrics were in good range: The difference between reference and estimated volumes were between -0.036 and 0.014 mL, the respective standard deviation was between 0.23 and 0.26 mL. For the diameters, the differences were between -0.137 and 0.011 mm and the standard deviations between 0.69 and 0.79 mm for all methods.

The optimal parameters averaged over all 1,000 validation runs were P1 = 272 ± 5 HU for absolute thresholding, P1 = 194 ± 17 HU and P2 = 0.29 ± 0.005 for the absolute thresholding with partial-volume correction, P1 = 0.48 ± 0.02 for the median based thresholding, and P1 = 0.43 ± 0.04 and P2 = 0.11 ± 0.08 for median based with partial-volume correction.

The Bartlett’s test indicated no significant difference in the distributions of the variances between reference and estimated values for both volume and diameter (p = 0.55 and p = 0.87, respectively).

Finally, for visual assessment, detailed volume-renderings of all uroliths are given in the Supporting Material.

## Discussion

In this study, we developed and compared four algorithms for segmentation of uroliths in computed tomography images. Our results show that all algorithms can be applied with a high diagnostic accuracy regarding determination of maximal diameter and volume. Further, no difference in performance could be detected between the algorithms tested. The developed algorithms therefore all enable for reliable segmentation of uroliths in low dose CT examinations.

The developed algorithms differed in the application of the cut-off value and the surface correction. Not being able to find a significant difference between the algorithms in our study allows for several possible interpretations. On the one hand, the fixed cut-off could already represent a statistically valid solution to the problem of partial-volume effects, which cannot be improved in the given system even by mathematically considering these effects. On the other hand, it is possible that the adaptive cut-off values and surface corrections implemented for the correction of the partial-volume effects could not achieve sufficient correction. Finally, it is possible that the differences are so minimal that they could not be detected in this study. In terms of deviation measures to the ground-truth, there are slight differences. In volume correlations, there seems to be a bias for uroliths in the range of 0.1–0.5 mL. An explanation for this could be the fact that the water-displacement-based volumes-measuring method included a change between measuring cylinders at several positions, one around 0.1 mL.

Our results are consistent with previously published studies in that we can also show that valid volumetry of uroliths can be realized with a simple Hounsfield unit cut-off value [7, 8]. Even though there is no other published comparison of different algorithms for segmentation of uroliths available so far, there seems to be no indication that a correction of interfacial effects would be necessary in this segmentation application.

Our work shows that the segmentation of uroliths already with a simple cut-off value leads to good results for the determination of maximum diameter and volume and that no improvement of the segmentation can be achieved by applying complex correction methods. This is important because a simple region growing algorithm with a fixed cut-off value is straightforward to implement. It can be expected that implementations by different software vendors will yield the same standardized values, which probably would not be the case for more complicated, or deep learning based methods. Our result is therefore the basis for a widespread use of volumetry in the diagnosis of urolithiasis. In addition, our algorithm is browser-based, which would allow the cross-site use e.g. for multicenter studies.

The results of this ex-vivo study might be useful also for advances in in-vivo segmentation. The task of robust and fully automated detection and segmentation of renal stones in an abdominal image in vivo can probably only be solved using deep learning techniques. Deep learning methods rely on reproducible and accurate training data. Our results and our generated dataset might be useful to create and calibrate ground-truth training sets. We therefore made the full dataset freely available to the community (see data availability section).

However, our study also has some limitations. First of all, we examined relatively large uroliths in this study, the smallest concrement measured slightly less than 5 mm. We chose this selection of uroliths because smaller uroliths often do not require interventional therapy, so that the detection of maximum diameter and volume in clinical routine should be of secondary interest. However, the smaller the uroliths are, the stronger the influence of the partial-volume effects on the volumetry. The results of this study can therefore not directly be applied to the analysis of uroliths smaller than 5 mm in size.

With the current treatment options in humans in our country, stones are fragmented before removal and fragments are not bigger than 5–6 mm diameter. For larger uroliths we therefore partly had to depend on animal stones. This explains the stone compositions, which differ from standard human compositions (more cystine and struvite stones, less calcium oxalate stones). However, since the contrast properties of these uroliths are relatively similar in CT [12], we anticipate that the results are transferable to a purely human urolith collective. Due to data protection issues the baseline characteristics of the patients could not been obtained from the donating stone center, but we believe this data would not change the interpretation of the results of this study.

Further, this is an ex-vivo study and in the phantom we used, the uroliths lie in water, which is clinically more similar to the situation of a bladder stone than that of a kidney or ureter stone. However, the difference in density values between the retroperitoneal structures and water should be negligible in relation to the high contrast to the uroliths themselves.

Another limitation is that the semi-automated procedure of stone segmentation was performed by only one radiologist. However, the exact seeding point made no difference at all to the segmentation for the relatively large stones used in this study, which means that repetition of the labeling by additional radiologists would not have changed the data at all and was therefore not considered necessary.

In summary, even a simple cut-off value allows for a valid segmentation and measurement of diameter and volume of uroliths larger than 4 mm in size. In these cases the application of correction algorithms for volumetry does not seem necessary.

## Supporting information

S1 Fig 3D characterisation of uroliths 1–40. Uroliths are ordered by increasing volume. All uroliths are shown together with the values for diameter, volume and median HU. Further, the gray-value histograms in HU units x10^3 are shown. Coloring of the uroliths corresponds to the median HU value of the uroliths, increasing from blue (low) to high (red). Hence, uroliths with low density are more blueish, while uroliths with high density are more reddish. Median HU values as a measure for stone density might help to infer information on their chemical composition. However, a detailed analysis with this respect was beyond the scope of the present work.
(TIF)

S2 Fig 3D characterisation of uroliths 41–80. Uroliths are ordered by increasing volume. All uroliths are shown together with the values for diameter, volume and median HU. Further, the gray-value histograms in HU units x10^3 are shown. Coloring of the uroliths corresponds to the median HU value of the uroliths, increasing from blue (low) to high (red). Hence, uroliths with low density are more blueish, while uroliths with high density are more reddish. Median HU values as a measure for stone density might help to infer information on their chemical composition. However, a detailed analysis with this respect was beyond the scope of the present work.
(TIF)

S3 Fig 3D characterisation of uroliths 81–101. Uroliths are ordered by increasing volume. All uroliths are shown together with the values for diameter, volume and median HU. Further, the gray-value histograms in HU units x10^3 are shown. Coloring of the uroliths corresponds to the median HU value of the uroliths, increasing from blue (low) to high (red). Hence, uroliths with low density are more blueish, while uroliths with high density are more reddish. Median HU values as a measure for stone density might help to infer information on their chemical composition. However, a detailed analysis with this respect was beyond the scope of the present work.
(TIF)
