# Supplementary figures and images for: Effect of surface-partial-volume correction and adaptive threshold on segmentation of uroliths in computed tomography
Source: PLoS One. 2023 Jun 23;18(6):e0286016. doi: 10.1371/journal.pone.0286016 (PMC10289361; doi:10.1371/journal.pone.0286016)

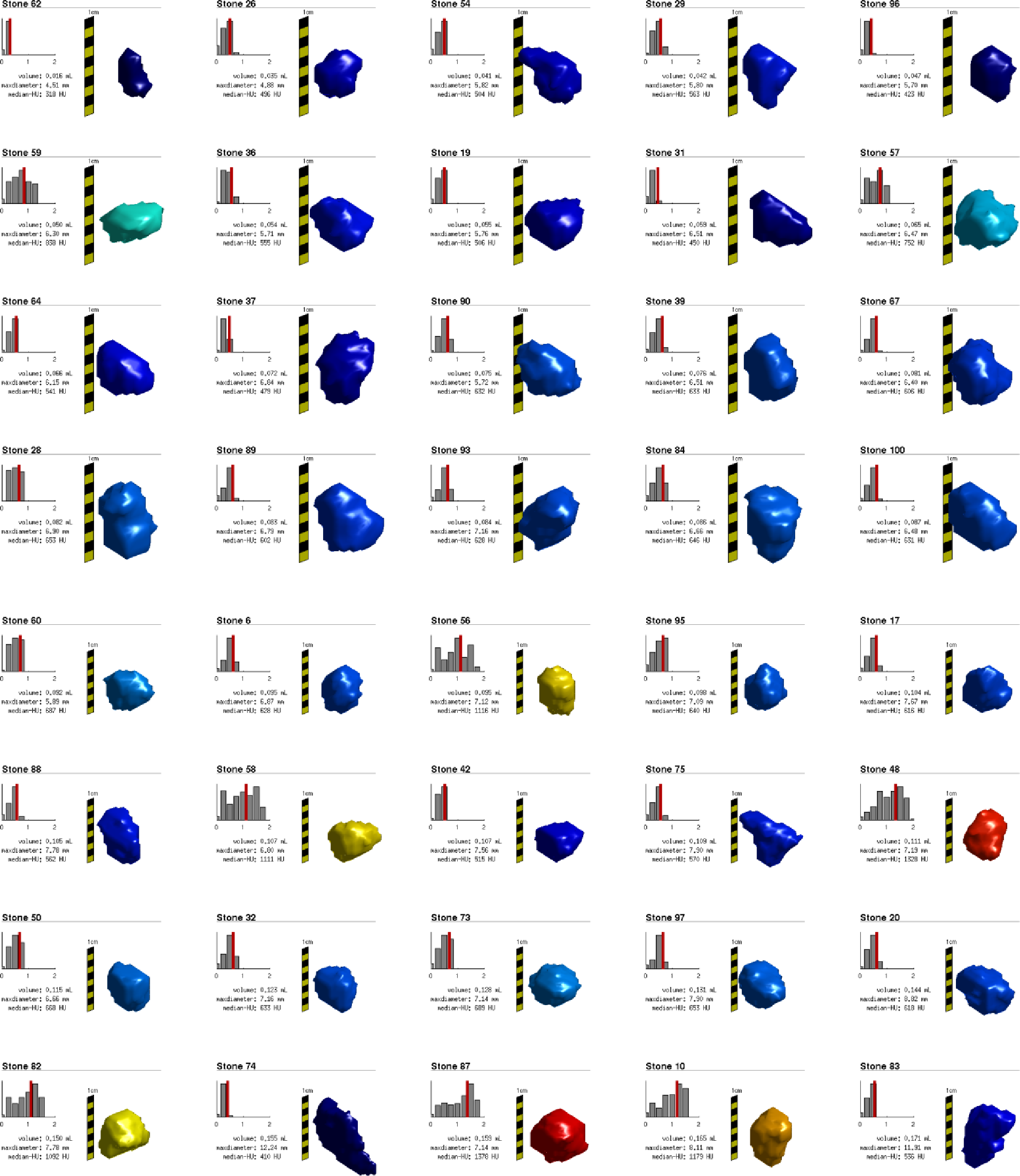

Supplement: S1 Fig — Uroliths are ordered by increasing volume. All uroliths are shown together with the values for diameter, volume and median HU. Further, the gray-value histograms in HU units x10^3 are shown. Coloring of the uroliths corresponds to the median HU value of the uroliths, increasing from blue (low) to high (red). Hence, uroliths with low density are more blueish, while uroliths with high density are more reddish. Median HU values as a measure for stone density might help to infer information on their chemical composition. However, a detailed analysis with this respect was beyond the scope of the present work. (TIF) [file pone.0286016.s001.tif]

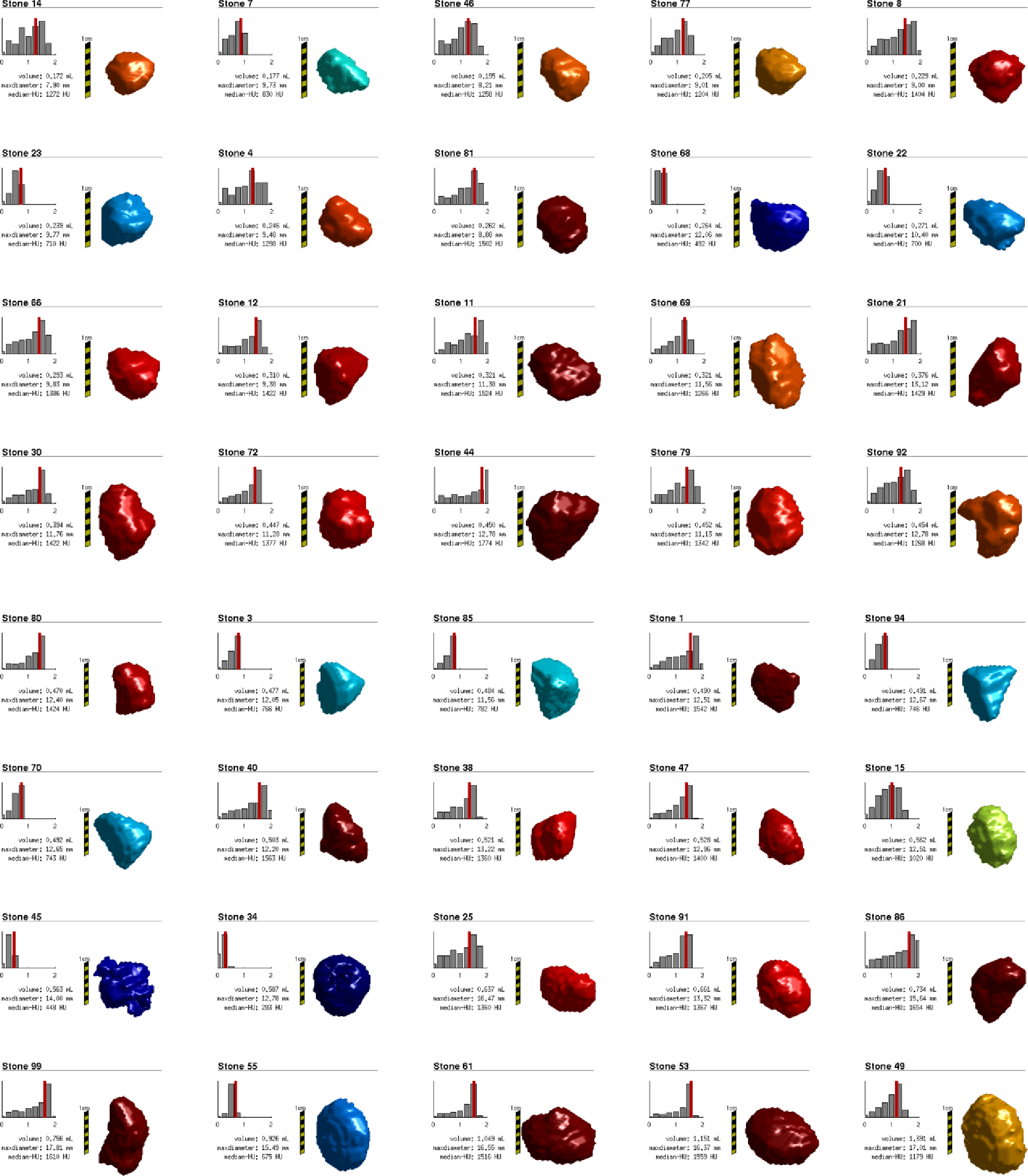

Supplement: S2 Fig — Uroliths are ordered by increasing volume. All uroliths are shown together with the values for diameter, volume and median HU. Further, the gray-value histograms in HU units x10^3 are shown. Coloring of the uroliths corresponds to the median HU value of the uroliths, increasing from blue (low) to high (red). Hence, uroliths with low density are more blueish, while uroliths with high density are more reddish. Median HU values as a measure for stone density might help to infer information on their chemical composition. However, a detailed analysis with this respect was beyond the scope of the present work. (TIF) [file pone.0286016.s002.tif]

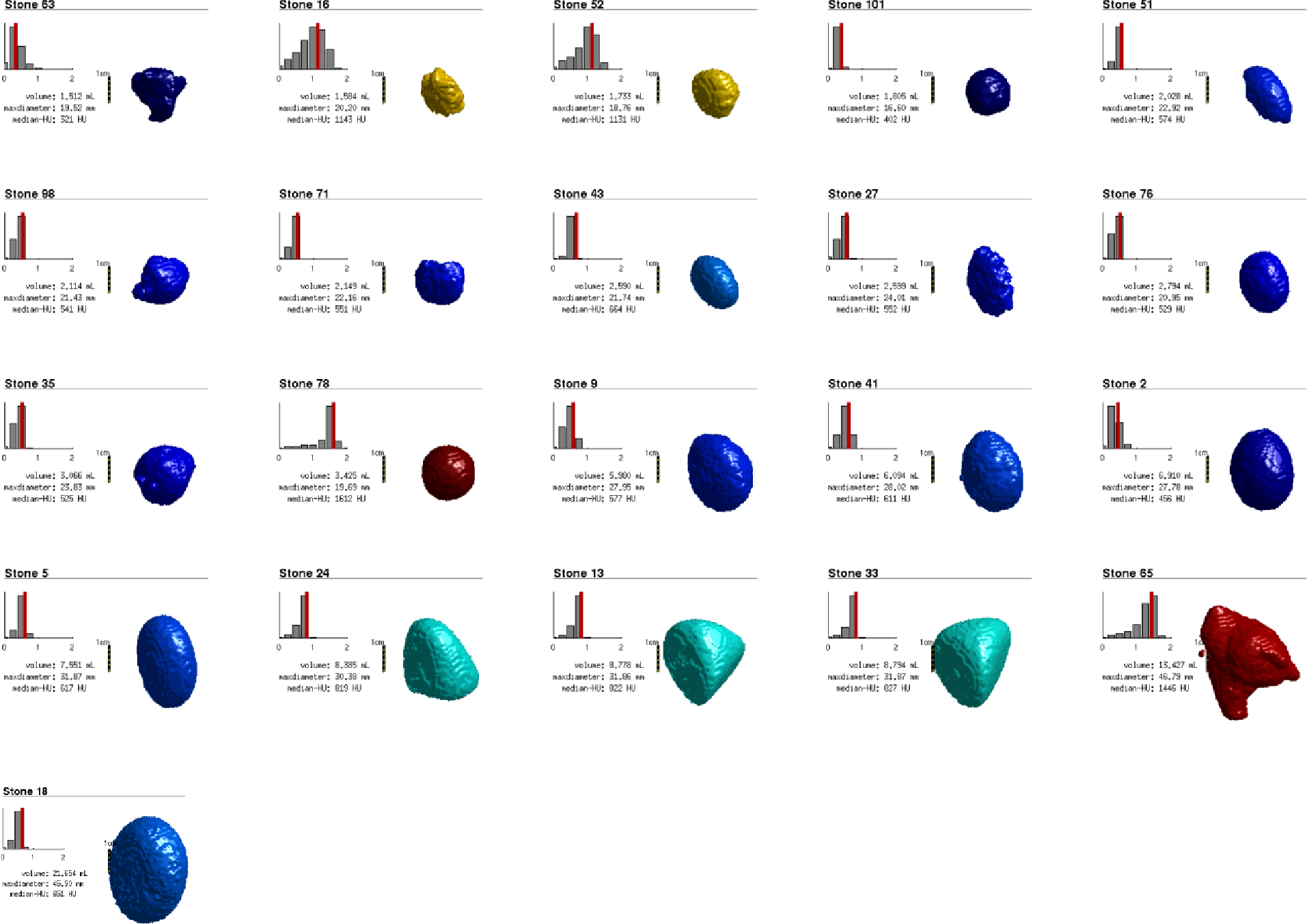

Supplement: S3 Fig — Uroliths are ordered by increasing volume. All uroliths are shown together with the values for diameter, volume and median HU. Further, the gray-value histograms in HU units x10^3 are shown. Coloring of the uroliths corresponds to the median HU value of the uroliths, increasing from blue (low) to high (red). Hence, uroliths with low density are more blueish, while uroliths with high density are more reddish. Median HU values as a measure for stone density might help to infer information on their chemical composition. However, a detailed analysis with this respect was beyond the scope of the present work. (TIF) [file pone.0286016.s003.tif]
